# The differential ability of asparagine and glutamine in promoting the closed/active enzyme conformation rationalizes the *Wolinella succinogenes* L-asparaginase substrate specificity

**DOI:** 10.1038/srep41643

**Published:** 2017-01-31

**Authors:** Hien Anh Nguyen, Donald L. Durden, Arnon Lavie

**Affiliations:** 1 The Jesse Brown VA Medical Center, Chicago, Illinois, United States of America; 2 Department of Biochemistry and Molecular Genetics, University of Illinois at Chicago, Chicago, Illinois, United States of America; 3 Department of Pediatrics, Division of Pediatric Hematology-Oncology, Moores Cancer Center, University of California San Diego Health System, La Jolla, California, United States of America

## Abstract

Many side effects of current FDA-approved L-asparaginases have been related to their secondary L-glutaminase activity. The *Wolinella succinogenes* L-asparaginase (*Wo*A) has been reported to be L-glutaminase free, suggesting it would have fewer side effects. Unexpectedly, the *Wo*A variant with a proline at position 121 (*Wo*A-P_121_) was found to have L-glutaminase activity in contrast to Uniprot entry P50286 (*Wo*A-S_121_) that has a serine residue at this position. Towards understanding how this residue impacts the L-glutaminase property, kinetic analysis was coupled with crystal structure determination of these *Wo*A variants. *Wo*A-S_121_ was confirmed to have much lower L-glutaminase activity than *Wo*A-P_121_, yet both showed comparable L-asparaginase activity. Structures of the *Wo*A variants in complex with L-aspartic acid versus L-glutamic acid provide insights into their differential substrate selectivity. Structural analysis suggests a mechanism by which residue 121 impacts the conformation of the conserved tyrosine 27, a component of the catalytically-important flexible N-terminal loop. Surprisingly, we could fully model this loop in either its open or closed conformations, revealing the roles of specific residues of an evolutionary conserved motif among this L-asparaginase family. Together, this work showcases critical residues that influence the ability of the flexible N-terminal loop for adopting its active conformation, thereby effecting substrate specificity.

One of the biggest challenges of cancer chemotherapy is to identify drugs that eliminate tumour cells while at the same time minimize off-target effects. One strategy to achieve this type of tissue selectivity is to identify the “Achilles heel” of cancer cells; that is, a trait that can be exploited to target the cancer cells but that is absent in non-cancerous cells. A successful clinical example of exploiting such an Achilles heel is present in the treatment of acute lymphoblastic leukemia (ALL). Since unlike normal tissue this type of blood cancer is incapable of synthetizing L-asparagine (Asn) (i.e. its Achilles heel), ALL cells are completely dependent on extracellular supplies of this amino acid^1^. By administering enzyme drugs called L-asparaginases, which act to quickly deplete Asn in the blood, this drug specifically impacts ALL cells, starving them to death while leaving normal cells largely intact.

The discovery of enzymes with L-asparagine hydrolytic activity can be traced first to Lang in 1904 followed by Clementi in 1922 who reported the first comprehensive study on the capacity of different tissues from different animal classes to break down Asn into L-aspartic acid (Asp) and ammonia and termed this activity L-asparaginase^2^. Among mammals examined by Clementi, the guinea pig appeared to be unique in possessing L-asparaginase activity in blood serum. The development of L-asparaginase as a cancer therapeutic starts with Kidd in 1953 who detected an anti-lymphoma effect of the guinea pig serum, which was later revealed by Broome to be related to its L-asparaginase activity^3^,^4^. This prompted the discovery of the *Escherichia coli* L-asparaginase (*Ec*A), an enzyme drug that has been the cornerstone of ALL therapies since the late 1970 s 5. In 2011 the *Erwinia chrysanthemi* L-asparaginase (*Er*A) was FDA-approved and joined the L-asparaginase treatment regimens^6^. Notably, these L-asparaginases harbour an L-glutaminase secondary activity, that is, a capacity to deaminate the amino acid L-glutamine (Gln). Whether this secondary activity has any clinical relevance is still being debated, but it is clear that many of the toxic side effects of L-asparaginase therapy can be attributed to the L-glutaminase activity^7^,^8^.

To reduce the L-glutaminase–related toxicity of L-asparaginase therapy, several groups focused on engineering *Ec*A and *Er*A towards low L-glutaminase variants^9^,^10^,^11^. Another approach is to search for enzymes that naturally have lower L-glutaminase activity^12^,^13^,^14^. In the late 1970s, Distasio *et al*. reported a “glutaminase-free” L-asparaginase variant from *Wolinella succinogenes (Wo*A, previously called *Vibrio succinogenes*)^15^,^16^. The same group compared *Wo*A to *Ec*A reporting that while *Ec*A showed pronounced immunosuppressive effects, the glutaminase-free *Wo*A did not suppress the *in vivo* immune response in mice and lacked hepatotoxicity^17^,^18^,^19^,^20^,^21^. Another group confirmed the lack of immunosuppression in the PEGylated version of *Wo*A versus PEG-*Ec*A^22^, suggesting *Wo*A is potentially a safer drug than *Ec*A, mostly due to its glutaminase-free characteristic. In 1996, Lubkowski *et al*. reported the first crystal structure of *Wo*A along with its primary amino acid sequence^23^. Between 2001–2008, *Wo*A was evaluated clinically through a US National Cancer Institute Rapid Access to Intervention Development (NCI RAID) grant. However, *Wo*A produced via this program was found to be toxic in patients and unexpectedly had significant L-glutaminase activity^10^. During that time, advance in genome sequencing shed light into the complete genome sequence of *Wolinella succinogenes* and an updated sequence of *Wo*A was deposited, revealing a serine in position 121 24, in conflict with a proline at this position as reported by Lubkowski^23^. Whether one of these two *Wo*A variants, *Wo*A-Ser121 or *Wo*A-Pro121 (denoted herein as *Wo*A-*S*_121_ and *Wo*A-P_121_, respectively) is truly “glutaminase-free” remained unanswered. We sought to clarify this question and to further explain the substrate discrimination, if there is any, between the two forms, by solving the crystal structures of both variants in complex with either the product Asp or L-glutamic acid (Glu). Whereas the first *Wo*A structure solved by Lubkowski (deposited on the Protein Data Bank PDB ID: 1WSA) was free of ligand and the catalytically important flexible N-terminal loop of ~20 amino acids was missing due to poor electron density, here we report four crystal structures (*Wo*A-S_121_ + Asp; *Wo*A-S_121_ + Glu; *Wo*A-P_121_ + Asp and *Wo*A-P_121_ + Glu) where we could model the entire protein sequence. Surprisingly, these structures also revealed two completely distinct enzyme states; either active/closed or inactive/open, of the N-terminal flexible loop. To our knowledge, the *Wo*A-S_121_ + Asp, *Wo*A-S_121_ + Glu and *Wo*A-P_121_ + Glu structures reported in this study represent the first L-asparaginase structures caught in its fully inactive/open state in the presence of product ligands, while the *Wo*A-P_121_ + Asp structure is the first closed/active state of *Wo*A reported so far. Visualizing the very different conformational states of the N-terminal loop allows us to decipher the roles of evolutionary conserved residues in this bacterial L-asparaginases family. Additionally, analysis of these structures provides a structural basis for the equally efficient L-asparaginase reaction of *Wo*A-S_121_ and *Wo*A-P_121_, yet the very different L-glutaminase reaction kinetics. Together, this work significantly increases our understanding of this important L-asparaginase enzyme family, providing deeper understanding of how these enzymes function, and how seemingly minor changes in sequence can dramatically impact their substrate specificity.

## Results

### A single point mutation determines substrate selectivity

Kinetic characterization of *Wo*A-P_121_ and *Wo*A-S_121_ showed very similar L-asparaginase parameters (Table 1) but strikingly different L-glutaminase activities (Table 2). Whether the residue at position 121 is a serine or a proline, the L-asparaginase efficiency, as indicated by the k_cat_/Km parameter (~4 sec^−1^μM^−1^), as well as the observed turn-over rates at the physiologically-relevant Asn concentration of 50 μM (~60 sec^−1^), are essentially identical. In contrast, the L-glutaminase rates at either saturated substrate concentration or at 0.5 mM – the physiological blood concentration of Gln – were ~8 fold higher in *Wo*A-P_121_ compared to *Wo*A-S_121_. Consequently, with the same L-asparaginase potency but higher L-glutaminase activity, *Wo*A-P_121_ is a less Asn-specific enzyme compared to *Wo*A-S_121_, both at the condition of saturating substrate concentration or the one reflecting the physiological (and hence the clinically relevant) Asn and Gln concentrations in blood, as presented in Table 3. Interestingly, while the L-glutaminase turnover rate is very sensitive to the nature of amino acid at position 121, the Michaelis constant for both Asn and Gln substrates is not affected (~20 μM for Asn and ~0.35 mM for Gln in both *Wo*A-S_121_ and *Wo*A-P_121_). This result suggests that residue 121 governs the Asn versus Gln selectivity via a mechanism different than substrate binding.

### *Wo*A-S_121_ and *Wo*A-P_121_ complex structures with Asp and Glu reveal the open and closed conformations of the N-terminal loop

To understand the molecular basis for the change in Asn/Gln selectivity between *Wo*A-S_121_ and *Wo*A-P_121,_ we crystallized and collected high-resolution diffraction data sets of these *Wo*A variants in complex with the products of the L-asparaginase and L-glutaminase reactions, Asp and Glu, respectively (see Table 4 for data collection and refinement statistics). *Wo*A, like most bacterial Type II L-asparaginases, is a tetramer built by a dimer-of-dimer with 330 residues per protomer. In our structures we observe that the overall fold of each protomer follows the general two-domain fold of bacterial type II L-asparaginases. Each of the four analysed complexes, *Wo*A-P_121_ + Asp, *Wo*A-P_121_ + Glu, *Wo*A-S_121_ + Asp and *Wo*A-S_121_ + Glu, has in the crystallographic asymmetric unit essentially four (or 8 in the case of *Wo*A-S_121_ + Glu) identical protomers that build up the corresponding tetramer(s), as shown by very small root mean square deviations of ~0.07 Å; 0.06 Å; 0.07 Å and 0.05 Å, respectively (over 300–330 atoms). The equivalency between protomers within a structure allows us to focus on a single protomer, or on the active dimer when needed, in the analysis that follows.

**Figure 1:**
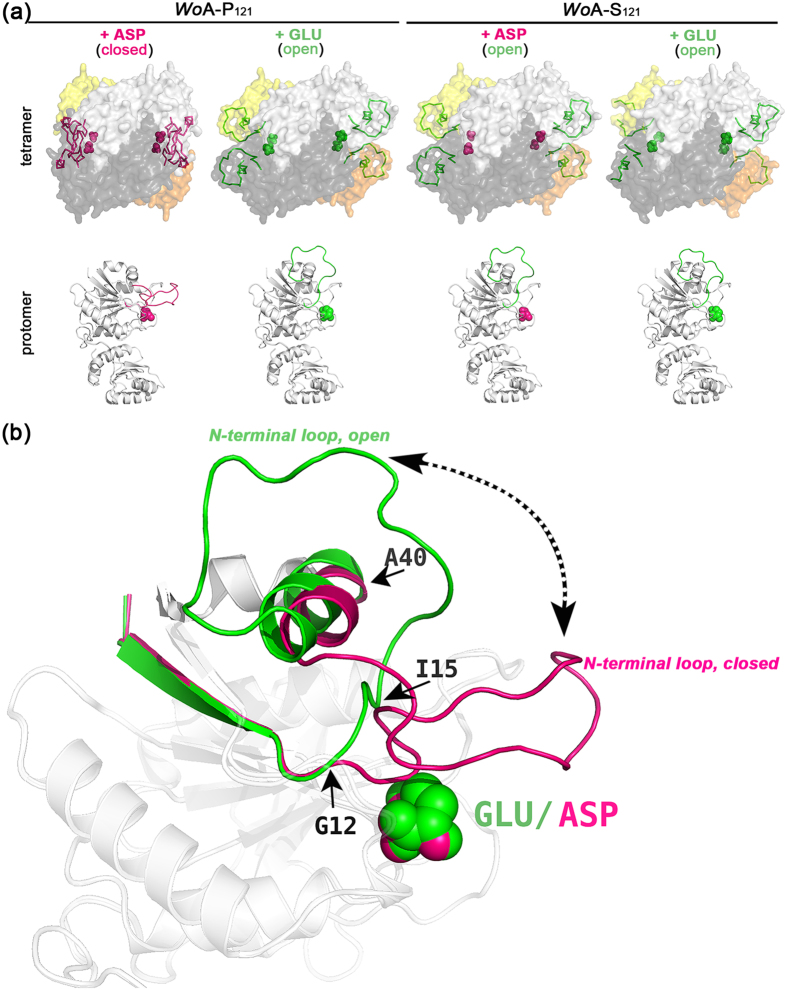
Visualizing the open and closed conformations of the flexible N-terminal loop. (**a**) Upper panel: Surface representation of the *Wo*A tetramer adopting the closed conformation in the *Wo*A-P_121_ + Asp structure and open conformation in the *Wo*A-P_121_ + Glu, *Wo*A-S_121_ + Asp, and *Wo*A-S_121_ + Glu structures. The dimer-of-dimers that build the tetramers are coloured in light and dark shades of grey and orange, respectively. Residues adopting the closed (and active) conformation of the N-terminal loop are highlighted in magenta, whereas those residues adopting the open (and inactive) loop conformation are shown in green. The ligand, Asp (magenta) or Glu (green) pinpoints the location of the four independent active sites. Lower panel: Cartoon representation of a single protomer (all 4 protomers are identical) from each of the structures in the upper panel highlighting the flexible N-terminal loop closing on the ligand in *Wo*A-P_121_ + Asp while opening further from the ligands in *Wo*A-P_121_ + Glu, *Wo*A-S_121_ + Asp, and *Wo*A-S_121_ + Glu structures. (**b**) Overlay of the N-terminal region of *Wo*A-P_121_ + Asp and *Wo*A-P_121_ + Glu showcasing the two conformations of the flexible loops (open, green and closed, magenta). The two states first split at Gly12 before re-joining at Ile15; then separating again towards or away from the ligand before ultimately re-joining at Ala40.

**Figure 2:**
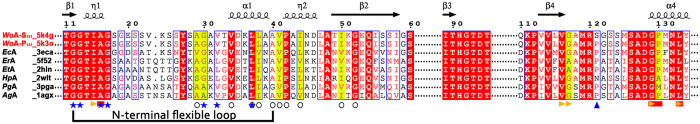
Sequence alignment of select bacterial L-asparaginases. Despite the key function of the flexible N-terminal loop (boundaries indicated by square bracket), amino acid sequence conservation is limited to the first segment, to the tyrosine that approaches the ligand (Tyr27 in *Wo*A), and several hydrophobic residues. In this alignment, the strictly and mostly conserved residues are highlighted in red and yellow, respectively. Open circles indicate conserved hydrophobic residues stabilizing the N-terminal loop in one or both conformations. Blue stars and orange arrows indicate conserved hydrophobic residues stabilizing the loop in its closed/active and open/inactive conformations, respectively. Red boxes indicate conserved hydrophobic backbone residues stabilizing the internal-hinge, Ile15 in *Wo*A. Blue arrow shows the amino acid at position 121^st^ in *Wo*A. The secondary structure shown above the primary sequence is based on the *Wo*A-P_121_ + Asp structure (PDB ID 5K3O). Numbering is based on WoA sequence. The UniProt entries of corresponding crystal structures used for this alignment are: *Wolinella succinogenes* L-asparaginase, *Wo*A-S_121_ + Asp_5K4G, P50286; *Escherichia coli* L-asparaginase, *Ec*A_3ECA, P00805; *Erwinia chrysanthemi* L-asparaginase, *Er*A_5F52, P06608; *Erwinia carotovora* L-asparaginase, *Et*A_2HLN, Q6Q4F4; *Helicobacter pylori* L-asparaginase, HpA_2WLT, O25424; *Pseudomonas sp.* L-asparaginase*, Pg*A_3PGA, P10182 and *Acinetobacter glutaminasificans* L-asparaginase*, Ag*A_1AGX, P10172.

The prime novelty of this report is the observation for the first time of two very distinct conformations for the flexible N-terminal loop of *Wo*A (residues 12–39). In all previously reported structures of bacterial type II L-asparaginases, the catalytically important N-terminal loop is either not observed, or if solved in the presence of ligand, is seen closed upon the active site with its conserved tyrosine residue in proximity to the ligand. Since all of the four structures presented here were solved in the presence of ligand, we expected the enzyme to adopt the closed N-terminal loop conformation in all structures. Indeed, this is what we observed in the *Wo*A-P_121_ + Asp structure (Fig. 1). Surprisingly, we observed unambiguous electron density for an open conformation of the flexible loop in the other three structures, *Wo*A-P_121_ + Glu, *Wo*A-S_121_ + Asp and *Wo*A-*S*_121_ + Glu (Fig. 1 and Figure S1). Starting from Gly12 – the first glycine residue from the conserved motif ^11^TGGTIAG^17^ (Fig. 2), the N-terminal loop in its closed/active conformation directs towards the bound ligand while this loop in its open/inactive conformation branches away from the ligand (Fig. 1b). The two conformations meet up at the conserved Ile15 residue then split again towards or away from the ligand, before finally re-joining at Ala40 (Fig. 1b).

All of these structures were solved using crystals grown in the absence of ligand that were then soaked with either Asp or Glu (see Methods). As reminder, the previously reported *Wo*A ligand-free structure showed no electron density for ~20 residues of the flexible N-terminal loop^23^. For *Wo*A-P_121_, soaking the crystal briefly in Asp solution was sufficient in closing the loop. In sharp contrast, the structure of *Wo*A-S_121_ crystals soaked in the same Asp solution, despite showing clear electron density for the Asp molecule (Figure S2), adopted the open loop conformation. Likewise, both *Wo*A variants soaked in a Glu solution had ligand bound (Figure S2) but the open loop conformation (Fig. 1 and Figure S1).

To verify that the observed novel open loop conformation is not an experimental artefact, we collected additional data sets on *Wo*A-S_121_ crystals soaked with Glu. These repeated data collections all clearly showed the bound Glu and the same open loop conformation. We also considered the possibility of crystal contacts affecting the loop conformation. The fact that all crystals solved in this study were grown in the same conditions yet displayed either open or closed conformation depending on soaked ligand argues that this loop is not held in a particular conformation by crystal contacts. Crystal packing analysis revealed no close contacts between loop residues (11–40) and asymmetric mates, arguing that crystal packing is not influencing the observed conformation of the N-terminal loop. To totally exclude the possibility that crystal contacts are affecting the open loop conformation (though the fact that the *Wo*A-P_121_ + ASP showed the closed loop argues against crystal contacts being a factor), instead of soaking in the ligands, we co-crystallized *Wo*A-S_121_ or *Wo*A-P_121_ with Glu and *Wo*A-S_121_ with Asp. Recapitulating the soaked-structures, and providing evidence for the lack of any influence of crystals contacts on the N-terminal loop conformation, we observed in these co-crystallized structures the ligand clearly bound at the active site, and the flexible N-terminal loop in the open state with clear electron density throughout. Moreover, in the *Wo*S_121_-Glu structure, we observed slight variations in the open loop conformation among the eight protomers of the asymmetric unit. Together, these observations argue that crystal contacts play no role in the open conformation adopted by the flexible N-terminal loop.

The difference between the *Wo*A variants and in the ability of the ligands to promote the active N-terminal loop conformation demonstrates two important principles. The first is that Asp is more efficient than Glu in inducing loop closure. Assuming that such a difference also exists for the substrates (i.e. Asn and Gln) this would be one factor that explains the higher L-asparaginase activity of these enzymes relative to their L-glutaminase activity. The second principle is the presence of an effect of the residue at position 121 on the preferred conformation of the N-terminal loop. It is tempting to speculate that the reduced L-glutaminase rate of *Wo*A-S_121_ compared to *Wo*A-P_121_ is due to the fact that loop closure on Gln is less efficient in the former. Structural evidence supporting this hypothesis is presented below.

### The additional methylene group present in Glu sterically prevents active loop closure

**Figure 3:**
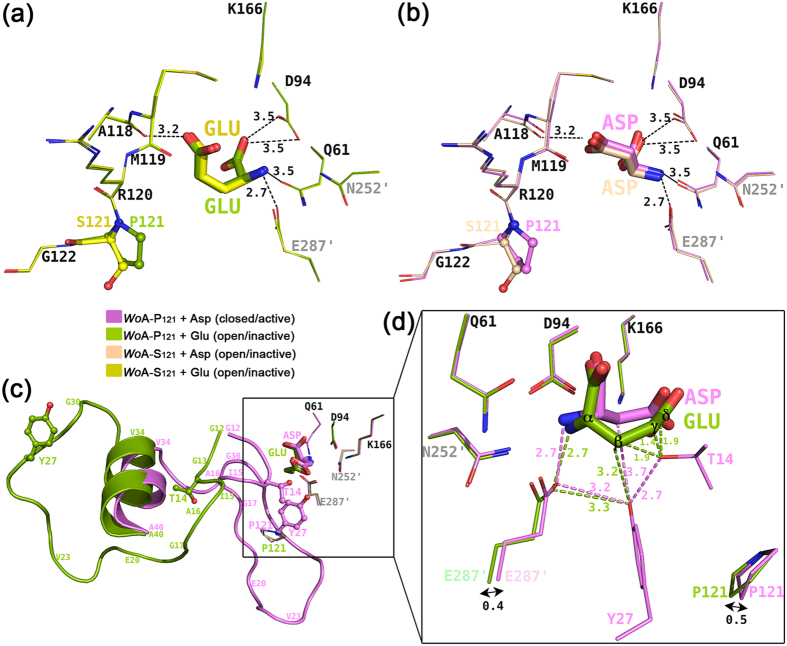
Substrate discrimination between GLN and ASN in *Wo*A-P_121_ and *Wo*A-S_121_. Overlay of (**a**) *Wo*A-P_121_ + Asp (pink) on top of *Wo*A-S_121_ + Asp (wheat) and (**b**) *Wo*A-P_121_ + Glu (green) on top of *Wo*A-S_121_ + Glu (yellow) showing essentially identical amino acid backbone environment near the active sites and identical ligand binding mode in each pair of overlaid structures. (**c**) Overlay of *Wo*A-P_121_ + Asp in close/active conformation (pink) where the active Thr14 and Tyr27 are in proximity of the ligand (stick representation); on top of *Wo*A-P_121_ + Glu in open/inactive conformation (green) where these two critical residues are facing outside of the active site. (**d**) Zoom of C. showcasing the steric hindrance of Glu on preventing loop closure, explaining Gln being a less favoured substrate than Asn. While the Asp fits without strain in the active site, the additional methylene group present in Glu challenges the closed conformation vis-à-vis Thr14 and Tyr27.

To understand the molecular mechanism underlying the substrate specificity, we analysed the binding mode of Asp and of Glu to the two *Wo*A variants. Overlay of *Wo*A-P_121_ + Glu and *Wo*A-S_121_ + Glu showed identical Glu binding mode (Fig. 3A). An identical Asp binding mode was also observed between the *Wo*A-P_121_ + Asp and *Wo*A-S_121_ + Asp structures, despite the former having the closed, and the latter the open, N-terminal loop conformation (Fig. 3B). The conclusions here are that the binding mode of the ligand (Asp or Glu) is not influenced by the nature of the residue at position 121 (serine or proline) or whether the N-terminal loop is in the open or closed states. Hence, for the purpose of understanding the differences in enzyme binding modes between Asp and Glu, we compared their binding to the *Wo*A-P_121_ variant, with any conclusions being also relevant to the *Wo*A-S_121_ variant.

In this comparison, *Wo*A-P_121_ + Asp represents the closed/active state of the enzyme (pink, Fig. 3C), whereas *Wo*A-P_121_ + Glu represents the open/inactive state (green, Fig. 3C). Both ligands fit with no steric clashes to the respective active sites. As the catalytically-relevant state is the one where the N-terminal loop is closed, to better understand how *Wo*A-P_121_ would accommodate Glu in its closed state, we modelled into the open *Wo*A-P_121_ + Glu structure the closed loop conformation as observed in the *Wo*A-P_121_ + Asp structure (Fig. 3D). This modelling reveals that whereas Asp fits without strain into the closed active site, binding of the larger ligand Glu (Glu has an extra methylene group relative to Asp) seems to be incompatible with loop closure due to several steric clashes. First, whilst the Asp Cβ atom adopts a conformation keeping it in a compatible distance (3.7 Å) from Tyr27, the Glu Cβ atom adopts the opposite conformation that would bring it a relatively short distance of ~3 Å to Tyr27 (Fig. 3D). Second, the active conformation taken by Thr14 in the *Wo*A-P_121_ + Asp closed structure is not compatible with neither the Cβ, Cγ nor Cδ atoms of Glu (modelled distances of ~1.4 to 1.9 Å).

Assuming that these structures with the products of the L-asparaginase and L-glutaminase reaction (Asp and Glu) provide a good approximation for the binding of the substrates (Asn and Gln), it is clear that for the enzyme to hydrolyse Gln (that is, adopt the closed conformation), either Gln adopts a different conformation to the one that we observe for Glu bound to the open state, or the enzyme state for Gln is different compared to the enzyme state with Asn, or a combination of the two adjustments. Since the Asp molecules in *Wo*A-P_121_ + Asp (active) and *Wo*A-S_121_ + Asp (inactive) occupied exactly same position (i.e. the conformation of the N-terminal loop has no effect on the Asp binding mode), we conclude that it is likely that the state of the N-terminal loop (open versus closed) would have little impact on the conformation adopted by Gln. So if the Gln does not adjust its conformation to be compatible with the closed state as seen in the complex with Asp, the only way for the enzyme to adopt the catalytic state is for the N-terminal loop to adjust its closed conformation to fit the larger Gln. With this assumption, we anticipate that several changes, especially at the Thr14 and Tyr27 positions, are required for the loop to close on Gln. Such a requirement for multiple structural adjustments to make the closed loop compatible with Gln binding correlates with kinetic data (Tables 1 and 2). Together, we attribute the impediment for loop closing on Gln but not Asn to be the main reason behind the L-glutaminase reaction rate being ~ eight times lower than the L-asparaginase reaction rate in *Wo*A-P_121_ and ~60-fold lower in case of *Wo*A-S_121_.

### Vital role of the interaction between Tyr27 and Pro121 in influencing substrate selectivity

**Figure 4:**
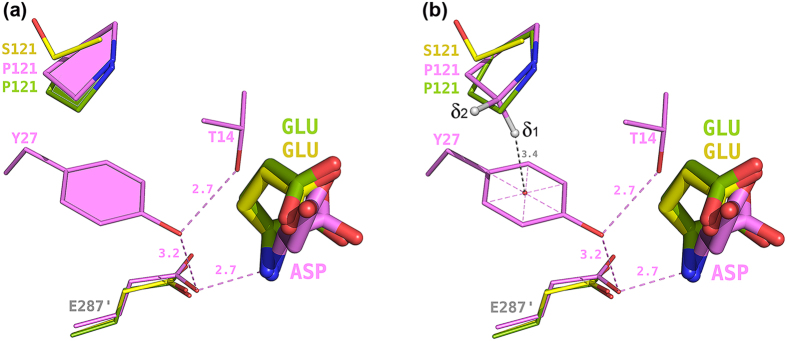
The presence of a proline at position 121 acts to stabilize the closed N-terminal loop conformation via interactions with Tyr27. These interactions include: (**a**) Hydrophobic ring-ring stacking interactions (illustrated by solid plans) and (**b**) CH/π interactions between hydrogen δ1 in Pro121 and the aromatic ring of Tyr27. The distance between Pro121-δ1 and the tyrosyl ring plan is ~3.4 Å. In contrast, the enzyme with a serine at this position (as in *Wo*A-S_121_) lacks this closed-state stabilizing interactions, rationalizing the increased relative propensity of *Wo*A-P_121_ for adopting the closed state.

We sought to answer the question why, while having very similar L-asparaginase potency, *Wo*A-S_121_ and *Wo*A-P_121_ behave strikingly differently when the substrate is Gln ? As discussed earlier, inspection of the *Wo*A-S_121_ + Glu and *Wo*A-P_121_ + Glu structures revealed essentially identical Glu conformations. The similar Gln Km values of these *Wo*A variants (Table 2), and assuming that Km ≈ Kd, suggests that a difference in substrate affinity can be excluded as the factor to explain the different Gln turnover rate between *Wo*A-S_121_ and *Wo*A-P_121_. We therefore extended our analysis to the ligand binding environments of *Wo*A-S_121_ + Glu versus that of *Wo*A-P_121_ + Glu. Except for the side chains of Ser121 versus Pro121, no notable differences were found in the active sites of the two structures (Fig. 3A). Since both the *Wo*A-S_121_ + Glu and *Wo*A-P_121_ + Glu structures are in the inactive/open conformations, as we have done previously to obtain an approximation of how the active/closed state would accommodate Glu, we modelled into these structures the active/closed N-terminal loop as seen in the *Wo*A-P_121_ + Asp structure (Fig. 4). As mentioned earlier, a steric clash between Thr14 and Glu occurs in this modelled structure. Nevertheless, if we assume that adjustments in the enzyme and in the Gln conformation solve this steric clash with Thr14, we can focus on the environment near residue 121, the residue that is modulating the substrate specificity. In such an analysis, our theoretical model shows that upon loop closure favourable interactions between Pro121 and Tyr27 would be made. Those interactions include in the first place a hydrophobic driving force (ring-ring stacking interactions illustrated by solid planes, Fig. 4A). Second, the two Cδ hydrogens Hδ1 and Hδ2 next to the prolyl nitrogen are electron-deficient protons, while the aromatic tyrosyl ring is rich in electron. In this case, as depicted in Fig. 4B, Hδ1 in Pro121 is ready to make CH/π interactions with the aromatic ring of Tyr27. Readers are invited to consult^25^,^26^ for more examples on CH/π interactions. In contrast, these interactions with Tyr27 are lost when the Pro121 is exchanged with a serine residue. The closing of the active loop on Gln in the *Wo*A-S_121_ variant therefore is relied solely on the hydrogen interaction between Tyr27 and the neighbouring Glu287′ (prime denotes a residue from a neighbouring protomer).

### Binding of Asp induces active loop closure in *Wo*A-P_121_ but not in *Wo*A-S_121_

**Figure 5:**
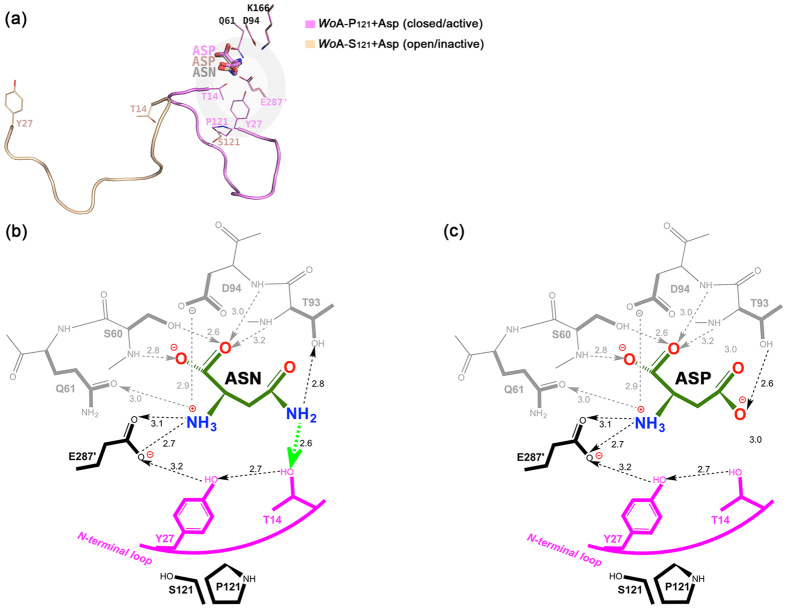
Rationalizing the identical L-asparaginase kinetic properties yet differential impact of Asp binding to *Wo*A-P_121_ and *Wo*A-S_121_. The complexes with Asp reveal a different conformation for *Wo*A-P_121_ (closed) and *Wo*A-S_121_ (open), yet the kinetics of Asn hydrolysis is identical for the two enzyme variants. (**a**) To understand the differential response to Asp and Asn, we modelled an Asn molecule (grey colour) into the active site of *Wo*A-S_121_ based on *Wo*A-S_121_ + Asp structure. Distinguishable hydrogen-bond networks for Asn (**b**) and Asp (**c**). When Asn is in the active site, strong hydrogen bonds between the substrate’s amide (proton donor) and the hydroxyl moieties of both Thr14 and Thr93 tether the loop to the closed conformation regardless if residue 121 is a proline or serine. When Asp is in the active site, the hydrogen network is broken between the ligand and Thr14, hindering loop closure. Only if proline is in position 121, it strengthens the closed-conformation of Tyr27 (see text), which in turn stabilizes Thr14 and that suffices to keep the loop in the closed state, explaining *Wo*A-P_121_ + Asp structure adopting the closed conformation. When serine replaces Pro121, the major stabilizing factor for Tyr27 is lost and it can no longer compensate the lack of bonding between the ligand and Thr14, resulting in the *Wo*A-S_121_ + Asp structure adopting the open conformation.

The kinetic results (Table 1) clearly show that *Wo*A-S_121_ and *Wo*A-P_121_ are essentially equivalent in their capacity to hydrolyse Asn. Thus it is logical to conclude that the efficiency of active loop closing on Asn for the *Wo*A-P_121_ and *Wo*A-S_121_ variants would be comparable. However, as previously mentioned, soaking a *Wo*A-P_121_ crystal briefly in an Asp solution was sufficient to close the N-terminal flexible loop (Fig. 5A, closed loop in pink), while multiple attempts to soak *Wo*A-S_121_ crystals for many days or even co-crystallization of the *Wo*A-S_121_ protein with Asp still resulted in the same Asp-bound open structure (Fig. 5A, open loop in wheat). Thus, ambiguity exists, where on the one hand kinetic studies detect no difference between the *Wo*A-S_121_ and *Wo*A-P_121_ variants in their ability to hydrolyse Asn, and on the other hand, crystallographic studies show the binding of the product Asp can close the loop if the amino acid at the 121^st^ position is a proline but not if it is a serine. These conflicting results suggest that the enzyme variants treat the substrate Asn and the product Asp differently. To understand this phenomena, and assuming that both the product and the substrate are bound at the exact same position, we docked a molecule of Asn in the active site of *Wo*A-S_121_ + Asp (Fig. 5A, grey) and asked the two following questions: (i) Why in the presence of Asn, both *Wo*A-S_121_ and *Wo*A-P_121_ can efficiently close the loop to execute L-asparaginase activity? and (ii) Why in the presence of Asp, the N-terminal loop readily closes on *Wo*A-P_121_ but not on *Wo*A-S_121_? The schematics showing the interactions made by Asn (Fig. 5B) and by Asp (Fig. 5C) show that both are held in the active site via networks of salt bridges and hydrogen bonds maintained by seven conserved catalytic residues Thr14, Tyr27, Ser60, Gln61, Thr93, Asp94 and Glu287′. In these schematics, the donor to acceptor relationship is denoted by the arrowhead direction. The difference between Asn and Asp is that the substrate’s amide nitrogen atom is a proton donor, while the product’s carboxylate is a proton acceptor. When the substrate Asn is in the active site (Fig. 5B), hydrogen bonds between the substrate’s amide and the hydroxyl moieties of both Thr14 and Thr93 tether the N-terminal loop to the closed conformation. In contrast, when the product Asp is in the active site (Fig. 5C), the hydrogen network between the ligand and Thr14 is broken, hindering loop closure. Intuitively, this difference between the substrate and product makes sense, since by the nature of the product losing an interaction that stabilizes the closed state, the enzyme becomes more prone to open, and in so doing, promote product release.

The above difference between Asp and Asn exists regardless of the identity of residue 121. However, the identity of residue 121 does influence the propensity of the enzyme to adopt the closed state. When residue 121 is a proline, it strengthens the closed-conformation of Tyr27 (as described earlier), which in turn stabilizes Thr14. In contrast, when serine replaces Pro121, a major stabilizing factor for Tyr27 is lost and it can no longer compensate for the lack of bonding between the ligand and Thr14, resulting in the *Wo*A-S_121_ + Asp structure adopting the open conformation. Hence, the extra interaction provided by Pro121 (but not by Ser121) suffices to promote loop closure, rationalizing our observation of the *Wo*A-P_121_ + Asp structure being in the closed conformation. This analysis recapitulates the vital role of the interaction between Pro121 and Tyr27 for the closing of the N-terminal loop.

### Conserved three-hinge arrangement of the N-terminal flexible loop among the L-asparaginases family

**Figure 6:**
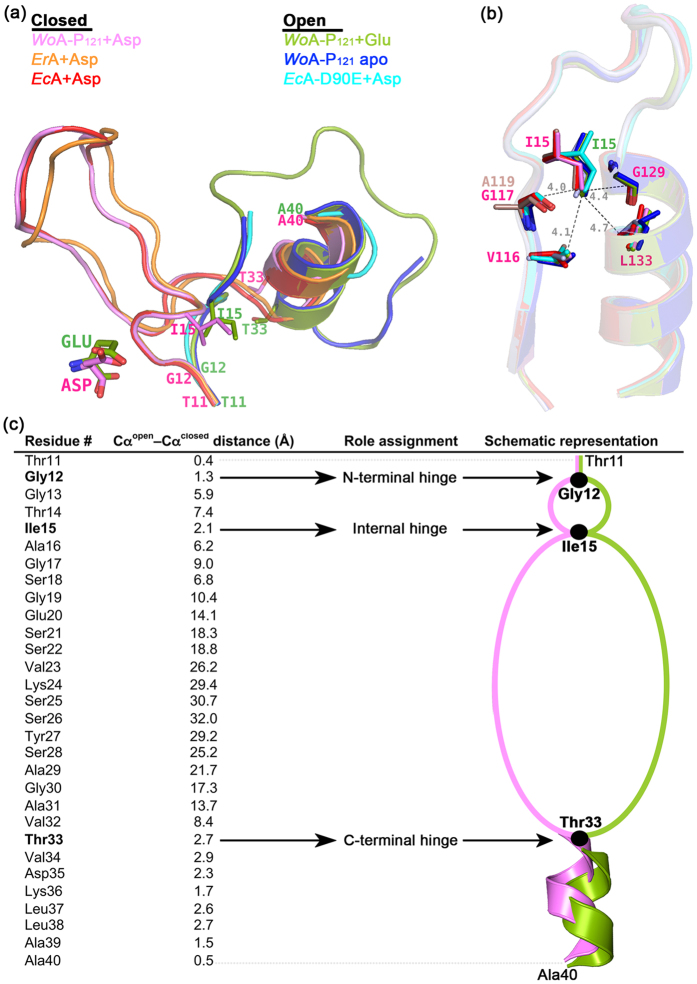
Conserved three-hinge structure of the N-terminal flexible loop among bacterial L-asparaginases family. (**a**) Overlay of the flexible N-terminal closed/active loops from *Wo*A-P_121_ + Asp (PDB ID: 5K3O, pink), *Er*A (5F52, orange) and *Ec*A (3ECA, red) on top of the flexible N-terminal open/inactive loops from *Wo*A-P_121_ + Glu (5K45, green), *Wo*A-P_121_ apo (1WSA, blue) and *Ec*A-D90E mutant (1IHD, cyan), revealing a conserved three-hinge model of the N-terminal flexible loop navigating between its active (closed) and inactive (open) conformations among bacterial L-asparaginases family. (**b**) Overlay of the structures used in A. revealed a strictly conserved hydrophobic pocket composing of conserved backbone residues stabilizing the central-hinge, Ile15 in *Wo*A. (**c**) Schematic representation of the three-hinge model in *Wo*A with Gly12, Ile15 and Thr33 being the N-terminal hinge, the internal hinge and the C-terminal hinge, respectively. The distance in angstroms between Cα of the N-terminal flexible loop residues (from residue 11 to 40) between their active/closed (pink) and inactive/open (green) conformations are reported.

The comparison between the open and closed conformations of the flexible N-terminal loop reveals that the change between the two states involves not two but rather three hinges. First, an N-terminal hinge at the *Wo*A Gly12 residue starts the different paths taken by the open and closed states (Fig. 6). Interestingly, these two paths converge after only three residues at Ile15, which we refer to as the Internal hinge. After converging at Ile15, the paths of the open and closed states immediately diverge again, only to unite at Thr33, a residue that acts as the C-terminal hinge. We also assign the helix immediately following the C-terminal hinge (residues Thr33 to Ala40) as a part of the flexible N-terminal loop, since it is shifted between the open and closed states. At Ala40 and beyond the enzyme is essentially identical between the open and closed states, showing that conformational changes between the open and closed states are limited to residues spanning the N-terminal loop (Gly12 to Ala39).

## Discussion

First, in this study we reported the first bacterial type II L-asparaginase structure showing the complete open state conformation in the presence of ligand. Bacterial type-II L-asparaginases (EC: 3.5.1.1) contain a catalytically-important N-terminal region. Whereas structures of these enzymes showcasing the closed loop state are abundantly available, the complete structure of the open-loop state has remained elusive. Partial understanding of the open-loop state was obtained in 1985, when Wlodawer *et al*.^27^ reported a preliminary crystallographic apo structure of *Wo*A (which was solved and modelled only 11 years later^23^) as an incomplete open structure, missing residues 19–26 of the N-terminal loop (PDB ID: **1WSA**, Fig. 6A, blue colour). Interestingly, those N-terminal loop residues that could be modelled in this apo structure adopted the open conformation we report here, suggesting that in the absence of ligand, the open conformation predominates. In 2001, several structures of the D90E mutant of the *E. coli* L-asparaginase were deposited (and later reported in 2014^28^) in which a segment of the open loop state was observed, but these structures were still missing loop residues 16–34 (PDB IDs: **1IHD**, **1JAZ** and **1JJA**). The exception was found at chain C of the last structure (PDB ID: 1JJA) where all loop residues were modelled to an open state. However, the authors of this work interpreted the observed open conformation of the N-terminal loop as an artefact due to crystal packing contacts^28^. When also considering the related glutaminase-asparaginase enzyme family (EC: 3.5.1.38), a structure of the enzyme from *Acinetobacter glutaminasificans* did present an open loop conformation (PDB ID: **3PGA**)^29^. However in this structure the electron density for the open loop was observed in only one of the four chains of the asymmetric unit and reported to have secondary structure, which was absent in the closed loop of the same enzyme (PDB ID: **4PGA**)^30^. Therefore, we believe that the *Wo*A-S_121_ + Asp, *Wo*A-S_121_ + Glu, and *Wo*P-S_121_ + Glu structures reported in this study represent the first L-asparaginase structures showing the entire physiologically-relevant open state conformation adopted by the flexible N-terminal loop. These structures allowed us to identify the structural elements that stabilize the open state conformation.

Secondly, in this report we propose a model of a structurally conserved three-hinge arrangement of the N-terminal flexible loop among the L-asparaginases family, with the *Wo*A Gly12, Ile15 and Thr33 being the N-terminal, the internal and the C-terminal hinges, respectively. The first two hinges belong to the strictly conserved consensus TGGTIAG motif present in bacterial Type II L-asparaginases (Fig. 2). Whereas the threonine at the centre of this motif has been known to be indispensable for the deamination reaction^31^,^32^,^33^, prior to this work the three residues before and after this catalytic threonine were of unassigned functions. When mutating the glycine adjacent to the catalytic threonine (*Ec*A Gly11, corresponding to *Wo*A Gly13) into either valine or leucine, Derst *et al*.^11^ found that the two mutated variants lost > 200 to ~3000 folds of L-asparaginase activity while the L-glutaminase became undetectable. These authors suggested that this second glycine is indispensable for the optimum orientation of the neighbouring catalytic side chains without further investigation. Our structures reveal that the first conserved glycine residue (*Wo*A Gly12) functions as the N-terminal hinge that allows the transition of the N-terminal loop either towards or away from the bound ligand (Fig. 6). Since only three residues later the open and closed states meet again, Gly13 functions to permit this tight turn. This explained the observation of Derst *et al*.^11^: when mutating the second glycine into either valine or leucine, the flexibility of the N-terminal loop is critically reduced, leading to a reduced function of the enzyme, in both of its L-asparaginase and L-glutaminase activities. Whereas this analysis ascribes to the two glycines that precede the catalytic threonine specific functions related to protein dynamics, the reason for strict conservation of the first threonine (*Wo*A Thr11) remains a mystery.

Regarding the residues that follow the catalytic threonine, immediately after it is an isoleucine^34^ (Fig. 2). Superposing our *Wo*A structures with abundantly available closed-state L-asparaginases and with limited previously described open-state structures shows that this isoleucine functions as an internal hinge (Fig. 6). The reason underlying the structurally conserved position of Ile15 is shown in the Fig. 6B, where this residue is trapped in the centre of a rigid and conserved hydrophobic pocket situated between strand β4 and helix α4. Even though between the open and closed states the change in position of the Ile15 Cα^open^ − Cα^closed^ is large (2.1 Å), the isoleucine side chain occupies nearly the same position, allowing it to keep the hydrophobic pocket intact (Fig. 6B). Hence, the conservation of Ile15 is due to the central role played by this residue in stabilizing the abovementioned hydrophobic pocket.

The final two residues of the TGGTIAG motif are an alanine and a glycine, corresponding to Ala16 and Gly17 in *Wo*A. Even though the environment in the immediate vicinity of Ala16 is vastly different between the open and closed states, in both a larger side chain would not fit (Figure S3). Whereas steric reasons rationalize the conservation of Ala16, for the following Gly17 we ascribe a role in loop dynamics, based on the observation that this residue undergoes a peptide flip when transitioning between the open and closed states.

In sum to this section, the highly conserved TGGTIAG motif not only contains an essential catalytic residue (the central threonine), but our work also shows that it contains two hinges (the N-terminal and Internal hinges) that facilitate the transition between the open and closed enzyme states. As expected from mobile regions, this motif includes three glycine residues, which impart the necessary flexibility to this part of the enzyme.

Finally, it is worth to highlight the role of CH/π-aromatic ring interactions and substrate specificity. Our kinetic results clearly demonstrate that *Wo*A-S_121_ (and not *Wo*A-P_121_) is the low L-glutaminase variant of *Wolinella succinogenes* L-asparaginase. Interestingly, this work reveals the connection between the propensity of the enzyme for adopting the closed state and its L-glutaminase activity. The mechanism behind this connection has to do with the central role of the conserved Tyr27 in *Wo*A for the loop closing event with either Gln or Asn. When the substrate Asn binds at the active site, loop closure is stabilized by the hydrogen bond network involving the substrate’s amide group, and the enzyme residues Thr14, Tyr27 and Glu287’. Importantly, this hydrogen network itself is sufficient for closed loop stabilization regardless of the nature residue 121. As a result, the L-asparaginase kinetic properties (k_cat_ and Km) of *Wo*A-S_121_ and *Wo*P-S_121_ are essentially identical (Table 1). However, when the larger substrate Gln occupies the active site, steric hindrance caused by the additional methylene group encumbers loop closure. It is for this substrate where the identity of the residue at position 121 becomes critical. Pro121 brings a ring stacking force along with π-aromatic ring interactions to stabilize Tyr27 in the closed state. In contrast, Ser121 fails to add any additional contribution to assist loop closing on Gln. As a result of the reduced ability of *Wo*A-S_121_ to achieve the closed/active state with Gln, the L-glutaminase rate (but not Km value) is reduced by ~8-fold related to *Wo*A-P_121_. In sum, this makes *Wo*A-S_121_ a more L-asparaginase specific enzyme than *Wo*A-P_121_.

How does this new understanding translate to the FDA approved L-asparaginases, *Ec*A and *Er*A? For *Ec*A, residues Thr12, Tyr25, Pro117 and Glu283′ occupy essentially the same positions as the *Wo*A residues Thr14, Tyr27, Pro121 and Glu287’, respectively. This suggests that the Thr-Tyr-Glu triad is operative also in *Ec*A to stabilize the closed state. Indeed, when *Ec*A Glu283’ is exchanged with either glutamine, glycine or valine (mutations that impair the Thr–Tyr–Glu triad), the *Ec*A L-asparaginase activity is diminished, presumably due to the reduced ability of the mutants for stabilizing the closed/active state^35^. Further evidence for an important role of the flexible loop tyrosine residue for the stabilization of the closed enzyme state comes from mutational studies of the *Ec*A tyrosine (Tyr25), where its replacement with alanine, histidine, phenylalanine or tryptophan, which disrupts either the hydrogen bonds network or the CH/π-aromatic ring interactions or both, reduced *k*_cat_ ~ 100 fold compared to the wild-type value^36^,^37^. Based on the garnered understanding of how Pro121 contributes to the stabilization of the closed state, for *Ec*A, this would predict that mutating Pro117 to a serine residue will negatively affect *Ec*A’s L-glutaminase but not its L-asparaginase activity. To test this prediction, we prepared the *Ec*A-P117S mutant and analysed its L-asparaginase and L-glutaminase activities. Indeed, as predicted, *Ec*A-P117S has increased specificity for Asn, with a ratio of L-asparaginase to L-glutaminase of ~100, compared to ~50 being this ratio for *Ec*A (Table 3). However, different to *Wo*A, where the P121S mutation has not detrimental effects on the L-asparaginase activity, for *Ec*A-P117S the L-asparaginase rate decreased from 44.1 to 12.6 sec^−1^. It is because this mutation had an even larger adverse effect on the L-glutaminase activity that in sum made *Ec*A-P117S a more Asn-selective enzyme.

The situation is very different for *Er*A. Despite having the corresponding *Ec*A residues (*Er*A Thr15, Tyr27, Pro123 and Glu289’) *Er*A residue Glu289′ has a completely different spatial position compared to *Wo*A Glu287′. As a result, *Er*A’s Glu289′ does not participate in the aforementioned triad hydrogen-bond network. By lacking the Tyr27–Glu289′ interaction, the catalytic positioning of *Er*A’s Tyr27 is maintained only by Thr15 and Pro123. Since *Er*A lacks such a functional triad, the presence of a proline (Pro123) at the analogous position to *Wo*A Pro121 becomes critical for *Er*A’s activity. Indeed, it was shown that mutating *Er*A’s Pro123 to either a serine or an asparagine residue abolishes the L-asparaginase activity^9^. This showcases the crucial Tyr–Pro CH/π-aromatic ring interactions for *Er*A in bringing the loop to the closed/active position.

In summary, this work reveals that the so-called “glutaminase-free” *Wo*A enzyme, the variant with a serine at position 121, has similar L-glutaminase activity as *Ec*A (k_cat_ ~ 0.89 and 1.58 sec^−1,^ respectively). In addition, we reveal how a proline at this position effects the L-glutaminase rate, but not the L-asparaginase rate. Additionally, for the first time we present the structure of the open flexible N-terminal loop in the presence of ligands, which serves to explain the function of several conserved residues in this L-asparaginase family. This understanding can inform the development of variants with more advantageous kinetic properties compared to the current FDA approved *Ec*A and *Er*A enzymes.

## Methods

### Gene Cloning and Mutagenesis

The genes of *Wo*A-S_121_ and *Wo*A-P_121_ were cloned into a pET28 vector. A PCR-based site-directed mutagenesis method was used to convert the Ser121 to Pro121 where the TCA codon corresponding to the nucleotides 363–365 in the ORF coding sequence within *Wo*A gene was converted to CCA. The entire ORF product sequence was confirmed by DNA sequencing.

### Protein Expression and Purification

The constructs carrying the genes of *Wo*A-S_121_ and *Wo*A-P_121_ were transformed into BL21-AI *E. coli.* A seed culture was incubated for 10 hours and used at OD_600nm_ of 1.0 to inoculate the production run. Protein expression was induced by IPTG and arabinose in 10-liter volume and harvested at an optimized OD. The corresponding proteins were purified to homogeneity under GMP conditions in the following sequence: SP-Sepharose XL chromatography; Q-Sepharose FF binding chromatography; tangential flow ultrafiltration and Q-Sepharose FF non-binding chromatography. The *Ec*A protein was expressed and purified as previously reported^9^,^34^.

### Kinetic Assays

L-asparaginase activity was determined using a continuous enzyme-coupled assay as previously described^9^,^34^,^38^,^39^. In brief, the assay measures the production of L-aspartate through the 1:1 oxidation of reduced NADH. NADH to NAD^+^ conversion was measured spectrophotometrically as a decrease in absorbance at 340 nm.

L-glutaminase activity determination was proceeded as previously reported^9^,^39^. In short, the enzymatic coupled assay measures the production of L-glutamate via the 1:1 formation of Iodonitrotetrazolium chloride (INT) – formazan. The conversion of INT to INT – formazan can be followed spectrophotometrically as an increase in absorbance at 500 nm.

All reactions were carried out at 37 °C and were triplicated. Rates were fit to the Michaelis-Menten equation using Sigma Plot software (Systat Software Inc).

### Crystallization, X-ray Data Collection, and Refinement

Crystals of the *Wo*A-S_121_ and *Wo*A-P_121_ were grown at 285 K using the hanging-drop vapour-diffusion method. 1 μL of enzyme at 10 mg/mL was mixed with 1 μL of reservoir buffer solution. For the co-crystallization experiment, 10 mM of L-aspartic acid sodium salt monohydrate (Sigma A6683) or 5 mM L-glutamic acid (Sigma 128420) was included into the enzyme solution. The difference in ligand concentrations was due to poor solubility of L-glutamic acid. The reservoir solution consisted of 0.1 M HEPES, pH 7.5 and 23% of PEG MME 2000.

Prior to data collection, apo crystals were soaked for 10 min with 10 mM L-aspartic acid or 5 mM L-glutamic acid in 0.1 M HEPES, pH 7.5 and 23% of PEG MME 2000. This step was skipped in crystals grown in the presence of ligands. Soaked crystals or ligand-bound crystals were then transferred to the same solutions respectively but supplemented with 25% of glycerol for cryoprotection.

Diffraction data were collected on the Life Sciences Collaborative Access Team (LS-CAT) beamlines 21-ID-F and 21-ID-G located at Argonne National Laboratory. Data were processed with the XDS package^40^. Structures were determined by molecular replacement with MOLREP^41^ using the structure (PDB entry 1WSA) as a search model. Refinement was conducted using REFMAC 5 42 and model building was conducted using Coot^43^. Data collection and refinement statistics are listed in Table 4. Structural figures were prepared using the PyMOL Molecular Graphics System (version 1.6.0, Schrödinger) and the ChemDraw Professional package (version 15.1).

## Additional Information

**Accession codes:** The atomic coordinates and structure factors were deposited to the Protein Data Bank with the ID codes: 5K3O; 5K45; 5K4G and 5K4H for *Wo*A-P_121_ + Asp, *Wo*A-P_121_ + Glu, *Wo*A-S_121_ + Asp and *Wo*A-S_121_ + Glu, respectively.

**How to cite this article:** Nguyen, H. A. *et al*. The differential ability of asparagine and glutamine in promoting the closed/active enzyme conformation rationalizes the *Wolinella succinogenes* L-asparaginase substrate specificity. *Sci. Rep.* **7**, 41643; doi: 10.1038/srep41643 (2017).

**Publisher's note:** Springer Nature remains neutral with regard to jurisdictional claims in published maps and institutional affiliations.

## Supplementary Material

Supplementary Data

## Figures and Tables

**Table 1:** L-asparaginase kinetic parameters for *Wo*A-S_121_ and *Wo*A-P_121._

Enzyme	k_cat_ (sec^−1^)	Km (μM)	k_cat_/Km (sec^−1^μM^−1^)	k_obs_@50 μM^a^ (sec^−1^)
*Wo*A-S_121_	97.8 ± 1.0	21.7 ± 1.2	4.5 ± 0.25	67.8 ± 0.4
*Wo*A-P_121_	86.7 ± 0.8	23.6 ± 1.1	3.7 ± 0.17	59.5 ± 0.8

^a^k_obs_@50 μM is the turnover rates at the physiologically concentration of Asn.

**Table 2:** L-glutaminase kinetic parameters for *Wo*A-S_121_ and *Wo*A-P_121._

Enzyme	k_cat_ (sec^−1^)	Km (mM)	k_cat_/Km (sec^−1^mM^−1^)	k_obs_@0.5 mM^a^ (sec^−1^)
*Wo*A-S_121_	1.58 ± 0.03	0.38 ± 0.05	4.2 ± 0.55	0.84 ± 0.01
*Wo*A-P_121_	11.33 ± 0.11	0.32 ± 0.02	35.3 ± 2.24	6.79 ± 0.08

^a^k_obs_@0.5 mM is the turnover rates at the physiologically concentration of Gln.

**Table 3:** Substrate selectivity of *Wo*S and *Wo*P.

Enzyme	L-asparaginase/L-glutaminase at saturating substrate concentration	L-asparaginase/L-glutaminase at physiological substrate concentration^a^
*Wo*A-S_121_	61.9	80.7
*Wo*A-P_121_	7.7	8.8
*Ec*A	49.9	N.D.
*Ec*A-P117S	103.4	N.D.

^a^L-asparaginase rate at 50 μM of Asn versus L-glutaminase rate at 0.5 mM of Gln.

N.D. Not determined.

**Table 4:** Data collection and refinement statistics.

Structure	*Wo*A*-*P_121_+Asp	*Wo*A*-*P_121_+Glu	*Wo*A*-*S_121_+Asp	*Wo*A*-*S_121_+Glu
PDB codes	5K3O	5K45	5K4G	5K4H
**Data collection statistics**				
X-ray source and detector	LS-CAT ID-FMARCCD 225	LS-CAT ID-MARCCD 225	LS-CAT ID-GMARCCD 300	LS-CAT ID-FMARCCD 225
Wavelength (Å)	0.97872	0.97872	0.97857	0.97872
Temperature (K)	100	100	100	100
Resolution^a^ (Å)	1.70 (1.80–1.70)	1.63 (1.72–1.63)	1.60 (1.70–1.60)	2.00 (2.11–2.00)
**Number of Reflections**				
Observed	522675 (82583)	577093 (74926)	522360 (83306)	294724(46009)
Unique	139440 (22086)	155380 (21426)	166478 (26952)	148378 (23525)
Completeness (%)	98.8 (97.2)	96.1 (82.5)	97.5 (98.2)	94.2 (92.6)
R_sym_ (%)	6.5 (71.8)	9.4 (77.7)	4.2 (42.9)	5.4 (49.4)
Average I/σ(I)	16.37 (2.17)	16.37 (2.17)	17.99 (3.17)	11.85 (1.69)
Space group	C 2	C 2	C 2	P 1
Unit cell (Å): a, b, c	144.76, 71.46, 141.50 90.00 118.07 90.00	145.81, 70.96, 142.28 90.00 118.08 90.00	146.09, 71.20, 142.60 90.00 118.00 90.00	63.54, 84.18, 120.55 87.22 77.76 70.84
Wilson B-factors (Å^2^)	21.3	27.9	19.3	29.0
**Refinement statistics**				
Refinement program	REFMAC5	REFMAC5	REFMAC5	REFMAC5
R_cryst_ (%)	17.54	16.26	16.63	18.10
R_free_ (%)	20.67	19.75	20.15	22.02
Resolution range (Å)	30.00–1.70	30.00–1.63	30.00–1.60	30.00–2.00
Protein molecules per a.u.	4	4	4	8
Number of atoms:				
Protein				
(ProtA, protB, protC, protD)	2452, 2452, 2452, 2452	2452, 2452, 2452, 2452	2450, 2450, 2450, 2450	2443, 2390, 2407, 2378
(ProtE, protF, protG, protH)	----, ----, ----, ----	----, ----, ----, ----	----, ----, ----, ----	2396, 2403, 2443, 2428
Water molecules	928	1485	1361	1567
Asp molecules	4	----	4	----
Glu molecules	----	4	----	8
R.m.s. deviation from ideal:				
Bond length (Å)	0.019	0.018	0.019	0.013
Bond angles (°)	1.78	1.75	1.89	1.58
Average B-factors (Å^2^)	26.9	28.3	26.9	36.9
Protein				
(ProtA, protB, protC, protD)	27.9, 27.0, 26.3, 24.2	28.4, 27.8, 26.4, 25.1	24.9, 24.1, 22.5, 20.5	35.8, 41.6, 37.5, 32.4
(ProtE, protF, protG, protH)	----, ----, ----, ----	----, ----, ----, ----	----, ----, ----, ----	35.0, 36.7, 40.8, 34.7
Water molecules	32.5	37.2	32.6	38.0
Asp molecules	25.4, 24.3, 27.6, 22.5	----, ----, ----, ----	30.3, 28.2, 32.1, 25.8	----, ----, ----, ----
Glu molecules	----, ----, ----, ----	37.4, 35.7, 35.7, 34.9	----, ----, ----, ----	43.9, 56.1, 44.9, 41.9
				47.5, 52.3, 50, 45.1
Ramachandran plot statistics (%)				
Most favored regions	96.45	96.36	96.97	96.11
Additionally allowed regions	2.63	2.77	2.40	3.26
Outlier regions	0.93	0.87	0.62	0.63

^a^High resolution shell in parenthesis; r.m.s., root-mean-square; a.u., asymmetric unit.
