# Supplementary material for: The differential ability of asparagine and glutamine in promoting the closed/active enzyme conformation rationalizes the Wolinella succinogenes L-asparaginase substrate specificity
Source: Sci Rep. 2017 Jan 31;7:41643. doi: 10.1038/srep41643 (PMC5282591; doi:10.1038/srep41643)
Supplement: Supplementary Data [file srep41643-s1.pdf]

Supplemental data

The differential ability of asparagine and glutamine in promoting the closed/active enzyme conformation rationalizes the *Wolinella succinogenes* L-asparaginase substrate specificity

Hien Anh Nguyen[1,2], Donald L. Durden[3,4] and Arnon Lavie[1,2,]*

[1]The Jesse Brown VA Medical Center, Chicago, Illinois, United States of America

[2]Department of Biochemistry and Molecular Genetics, University of Illinois at Chicago, Chicago, Illinois, United States of America

[3]Department of Pediatrics, Division of Pediatric Hematology-Oncology, Moores Cancer Center, University of California San Diego Health System, La Jolla, California, United States of America.

Running title: Bacterial L-ASNases N-terminal loop flexibility and *Wolinella succinogenes* L-ASNase Substrate specificity

*Address correspondence to: Arnon Lavie, PhD, 900 South Ashland Avenue, MBRB room 1108, Chicago, IL, 60607. Phone: (312) 355-5029; Fax: (312) 355-4535; E-mail Lavie@uic.edu

Figure S1

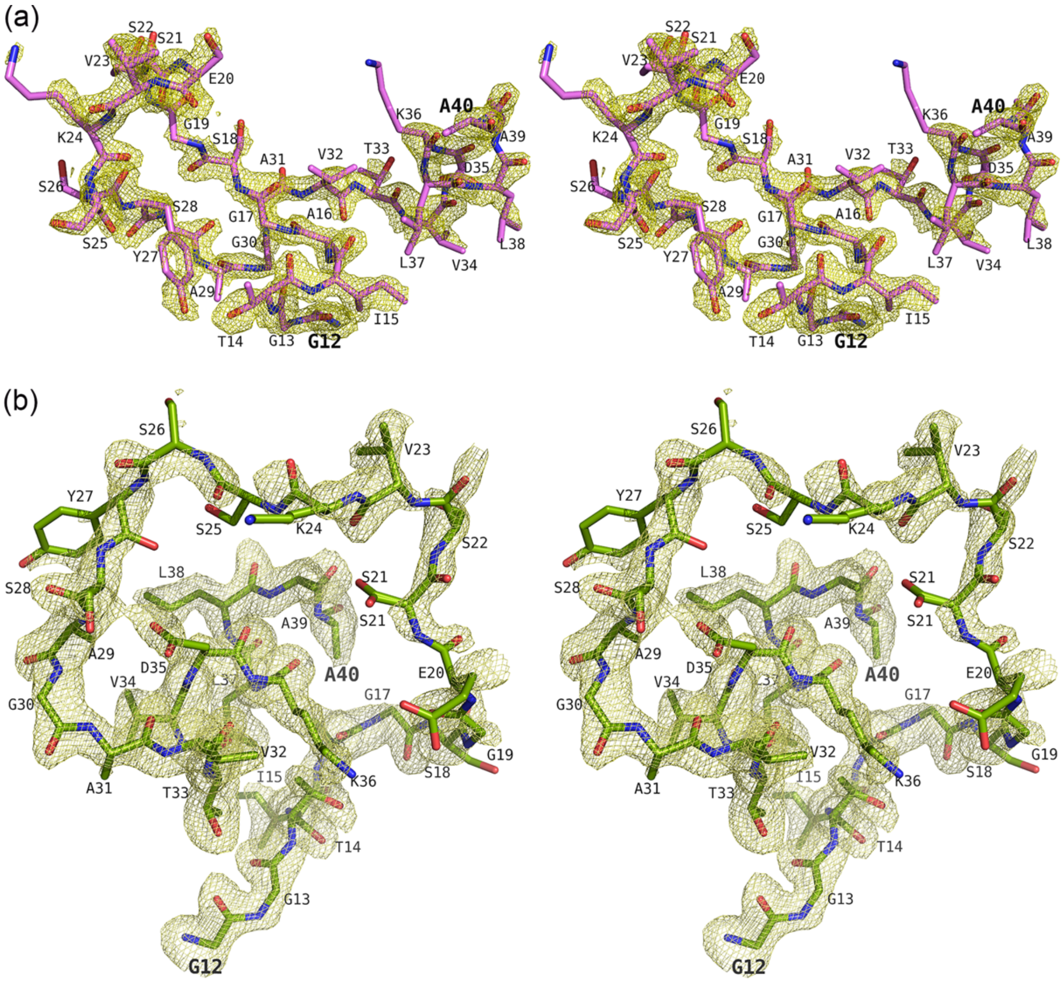

N-terminal Loop annealing omit maps. Omit density, overlaid on the final model, displayed in steric view for the flexible N-terminal loop as seen in **A.** *Wo*A-P_{121}+ASP (closed-state), chain A (pink) and **B.** *Wo*A-P_{121}+GLU (open state), chain A (green). Map shown is a simulated annealing omit map, contoured at 2.5 sigma, where in addition to the simulated annealing procedure, the loop region containing residues 12 – 40 was not included in the model.

Figure S2

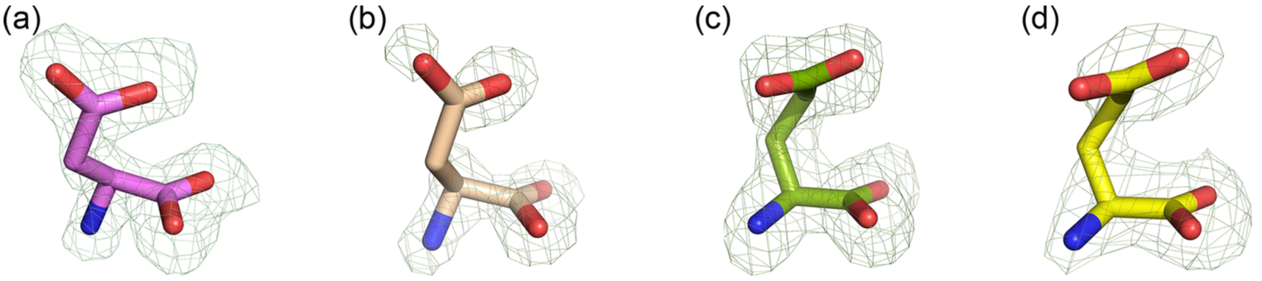

Ligands annealing omit maps. Omit density for the ligands as seen in **A.** *Wo*A-P_{121}+ASP (pink). **B.** *Wo*A-S_{121}+ASP (wheat). **C.** *Wo*A-P_{121}+GLU (green) and **D.** *Wo*A-S_{121}+GLU (yellow). Map shown is a simulated annealing omit map, contoured at 3.0 sigma, where the ligand was not included in the model.

Figure S3

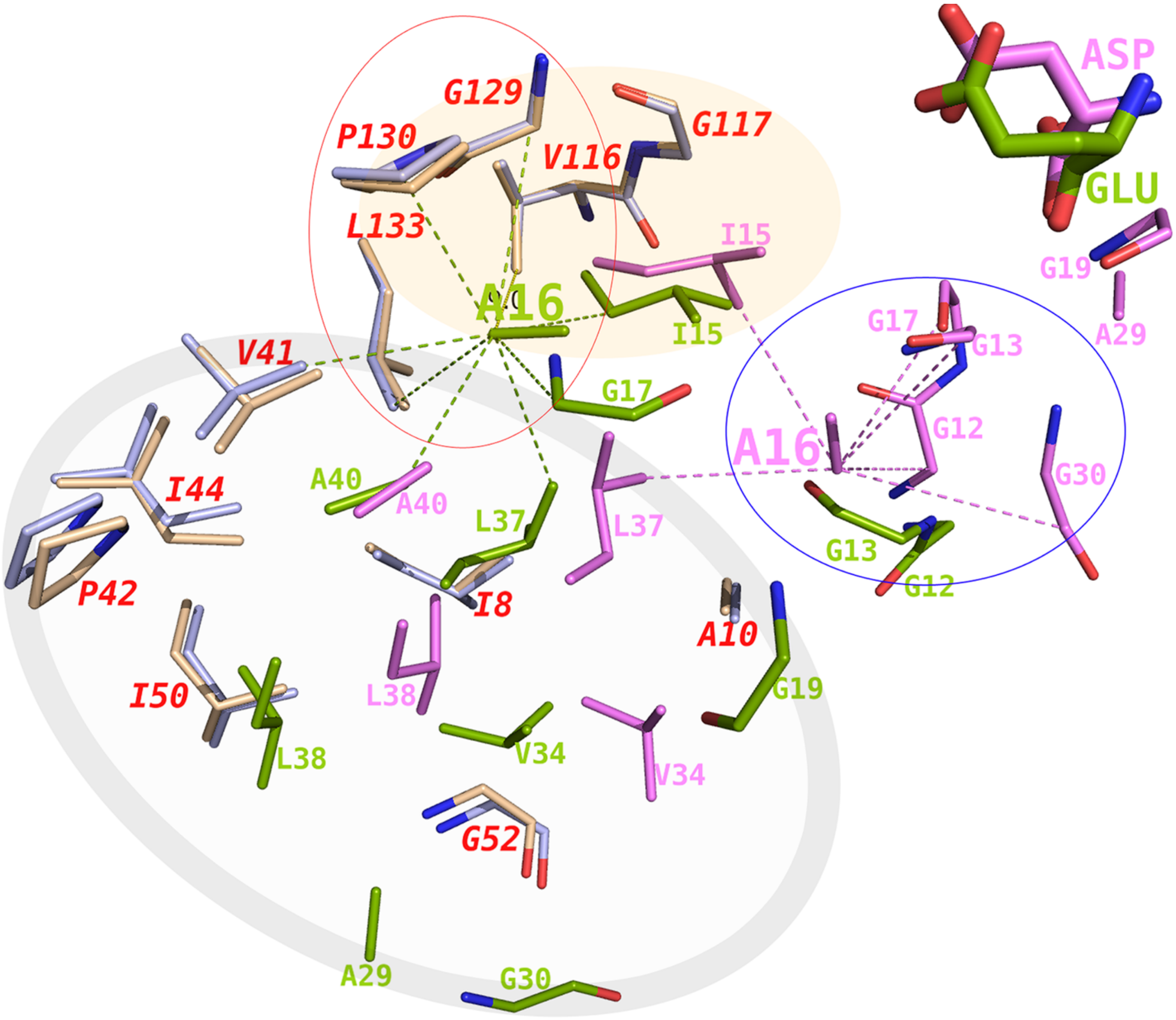

Hydrophobic networks of conserved residues stabilizing the N-terminal flexible loop. Overlay of the *Wo*A-P_{121}+Asp (close/active) and *Wo*A-P_{121}+Glu (open/inactive) structures where hydrophobic residues that are conserved among bacterial L-asparaginase type II are presented. Loop regions are highlighted in pink (closed) or green (open). Grey circle covering Ile15-Ala16-Gly17-Leu37-Ala40-Val41-Val116 stabilizes the loop in the open conformation. Blue circle with Gly12-Gly13-Ile15-Ala16-Gly17-Gly30-Leu37 stabilizes the loop in the closed conformation. Solid light orange and red circles depict conserved hydrophobic residues belonging to the rigid core of the protein and stabilize the central hinge Ile15.
